# Human T Cell Crosstalk Is Induced by Tumor Membrane Transfer

**DOI:** 10.1371/journal.pone.0118244

**Published:** 2015-02-11

**Authors:** Ronny Uzana, Galit Eisenberg, Sharon Merims, Shoshana Frankenburg, Aviad Pato, Eitan Yefenof, Roni Engelstein, Tamar Peretz, Arthur Machlenkin, Michal Lotem

**Affiliations:** 1 Sharett Institute of Oncology, Hadassah Medical Organization, P.O. Box 12000, Jerusalem 91120, Israel; 2 Lautenberg Center for General and Tumor Immunology, Hebrew University of Jerusalem, Jerusalem 91120, Israel

## Abstract

Trogocytosis is a contact-dependent unidirectional transfer of membrane fragments between immune effector cells and their targets, initially detected in T cells following interaction with professional antigen presenting cells (APC). Previously, we have demonstrated that trogocytosis also takes place between melanoma-specific cytotoxic T lymphocytes (CTLs) and their cognate tumors. In the present study, we took this finding a step further, focusing on the ability of melanoma membrane-imprinted CD8^+^ T cells to act as APCs (CD8^+^T-APCs). We demonstrate that, following trogocytosis, CD8^+^T-APCs directly present a variety of melanoma derived peptides to fraternal T cells with the same TCR specificity or to T cells with different TCRs. The resulting T cell-T cell immune synapse leads to (1) Activation of effector CTLs, as determined by proliferation, cytokine secretion and degranulation; (2) Fratricide (killing) of CD8^+^T-APCs by the activated CTLs. Thus, trogocytosis enables cross-reactivity among CD8^+^ T cells with interchanging roles of effectors and APCs. This dual function of tumor-reactive CTLs may hint at their ability to amplify or restrict reactivity against the tumor and participate in modulation of the anti-cancer immune response.

## Introduction

T cell activation requires the formation of an immunological synapse at the contact site of the lymphocyte with an antigen presenting cell (APC). Once the immunological synapse has been formed, it enables unidirectional transfer of membrane fragments from the APC to the effector T cell, a process named *trogocytosis* [1,2]. Transfer of biologic material from APCs to lymphocytes via cell to cell contact has been first published in 1973 [3]. Since then, numerous studies have shown the transfer of membrane components between immune cells, including NK, B and T lymphocytes, dendritic cells and monocytes [4,5],[6–9]. In addition to the morphological cell surface changes conferred by trogocytosis, functional properties, endowed by the transferred molecules, were also acquired by the recipient cell [9,10]. The multiplicity of activating and inhibitory molecules acquired by immune synapse-driven trogocytosis fits the emerging concept of immune plasticity, which underscores the ability of immune cells to modulate their function by using molecules they are not programmed to express [11,12]. An important feature of the immune system is its quick adaptation to variable external threats. In this context, trogocytosis provides an excellent tool for triggering fast secondary antigen presentation by the recipient cell. For instance, bystander dendritic cells (DCs), which capture membrane fragments from virus-infected DCs, present the acquired virus-derived pMHC and elicit a CD8^+^ T cell response, without being infected by the virus [13]. Similarly, activated B cells donate their Ig receptor and its antigenic specificity to bystander B cells, thus bestowing upon them the ability to present a non-cognate antigen to CD4^+^T cells [14].

As for T cells, the CD4^+^ subset acquired regulatory functions following the acquisition of MHC class II molecules from DCs and NK cells [15,16]. These CD4^+^ T cells reduced the recruitment of other CD4^+^ T cell subsets by a suppressive effect or through the induction of apoptosis [17]. Whereas the above studies linked secondary antigen presentation by T cells (T-APC) with inhibition of lymphocyte function, other studies demonstrated activation of effector lymphocytes [18–20]. It is conceivable that stimulatory *or* suppressive effects of T-APCs can be attained, depending on the CD4^+^ T cell subset involved [21]. Regarding CD8^+^ lymphocytes, the ability of CTLs to act as T-APC in vivo has been demonstrated in a transgenic mouse model [22].

We and others have reported that cancer cells donate membrane fragments containing tumor antigens to cognate T cells [23,24], and that the extent of trogocytosis correlates with the anti-tumor reactivity generated in T cell clones [25]. Recently, we demonstrated that tumor cell interaction with cytotoxic T lymphocytes (CTLs) yields a CTL subset imprinted with multiple tumor antigens [26]. In the present study we sought to investigate the role of melanoma antigen-imprinted CTLs in secondary antigen presentation. We show that, following trogocytosis, anti-tumor CD8^+^ T lymphocytes become an antigen presenting entity, denoted “CD8^+^T-APC”. Presentation of tumor-specific pMHC complexes by CD8^+^T-APC leads to intra- and inter-clonal CTL activation. Furthermore, we show that CD8^+^T-APC become targets for fratricide by tumor-specific CTLs. Our data suggest a novel role for CTLs in anti-cancer immunity and highlight their potential involvement in immunomodulation through secondary antigen presentation.

## Methods

### Mice and human cell cultures

OT-I T cell receptor (Va2/Vh5) transgenic mice, encoding a T cell receptor specific for the ovalbumin epitope (SIINFEKL-H-2Kb) were obtained from Dr. Avihai Hovav (Hebrew University, Jerusalem, Israel). Mice (female, 8–10 weeks) were maintained under specific pathogen-free conditions and sacrificed by CO_2_ asphyxiation. Then spleen cells were harvested and used for in-vitro studies. All experiments were conducted in strict accordance with Hadassah-Hebrew University Animal Facility and NIH guidelines (Approval number MD-10–12520–5, the authority for biological and biomedical models) and all efforts were made to minimize suffering. EG7 is a murine EL4 thymoma cell line of C57BL/6 origin, transfected with the *Ovalbumin* gene. EG7 cells were maintained in CM supplemented with 1 mg/mL geneticin (Life Technologies). Human melanoma cell line M171 (HLA-A2 negative) was established at the Sharett Institute of Oncology, Hadassah Medical Organization (Jerusalem, Israel). Generation of human melanoma cell lines was approved by the Institutional Review Board (Hadassah Medical organization IRB, Approval number 395–16.09.05), and all patients gave their written informed consent prior to initiation of cell cultures. 624*mel* (HLA-A2^+^) was a gift from M. Parkhurst (Surgery Branch, National Institutes of Health, Bethesda, MD). The expression of MART-1 and gp100 was confirmed by immunostaining using A-103 and HMB-45 mAbs, respectively (Dako). All cell lines were cultured in complete medium (CM) consisting of RPMI 1640, 25 mmol/L HEPES, 2 mmol/L L-glutamine and combined antibiotics (all from Life Technologies), supplemented with 10% heat-inactivated fetal calf serum. PBMCs were obtained from melanoma patients by cytopheresis, followed by centrifugation on Ficoll-Paque Plus gradient (Amersham, Uppsala, Sweden). Cells were cryo-preserved for future use. Tumor infiltrating lymphocyte (TIL) microcultures were initiated and expanded from tumor specimens taken from resected metastases of melanoma patients, as described [27]. Human lymphocytes were cultured in CM supplemented with 10% heat-inactivated human AB serum and 6000 IU/ml recombinant human IL-2 (rhIL-2; Chiron, Amsterdam, The Netherlands). Generation of human lymphocyte cell lines was approved by the Institutional Review Board (Hadassah Medical organization IRB, Approval number 395–16.09.05), and all patients gave their written informed consent prior to initiation of cell cultures.

### T cell clones and lines

MART-1_26–35_/HLA-A2-specific T cell clones 2E2, 2C7 and 2D11were generated from TIL cultures [25]. The gp100_154–162_/HLA-A2-specific 1G2 clone was established in a similar manner. Briefly, on day 14 of TIL initiation, the lymphocytes were stained with allophycocyanin-conjugated mouse anti-human CD8 mAb (eBioscience) and FITC-conjugated HLA-A*0201/gp100_154–162_ or FITC-conjugated HLA-A*0201/MART-_126–35_ dextramers (Immudex, Copenhagen, Denmark), according to the manufacturer’s protocol. CD8^+^dextramer^+^ cells were sorted using a BD FACSAria and directly cloned at one or two cells per well in 96-well plates in the presence of 30 ng/ml Ortho anti-CD3 (eBioscience), 6000 IU/ml rhIL-2, and 4 Gy-irradiated 5 × 10^4^ allogeneic PBMCs as feeder cells. Five days later, 6000 IU/ml rhIL-2 was added and renewed every 2 days thereafter. On day 14, the clone was assayed for IFN-γ secretion in a peptide-specific manner using commercially available ELISA reagents (R&D Systems, Minneapolis, MN). The reactive clones were further expanded in a second-round exposure to 30 ng/ml Ortho anti-CD3 and 6000 IU/ml rhIL-2 in the presence of 50-fold excess irradiated feeder cells. Dextramer staining and TCR β chain spectra-typing were used to confirm T cell clonality. The MUC-1_63–71_/HLA-A2-specific T cell line was generated from PBMC obtained from a melanoma patient [23]. S1 Table summarizes data on lymphoid cell lines and clones used in this work.

### Generation of CD8^+^T-APC and their use as targets for effector CTL

Tumor antigen-specific T cells were co-cultured with HLA-A2^+^ or HLA-A2^-^ melanoma cells (1–2 × 10^6^ lymphocytes/well in 200 microliter final volume) in U-shaped 96-well plates at a 1:1 effector-to-target ratio. Following incubation for 1 hour at 37°C, CD8^+^ T cells were purified using BD IMag anti-human CD8 particles (BD Biosciences). Sorted CD8^+^ T cells, designated CD8^+^T-APC, were labeled with the fluorescent lipophilic dye DiIC18 (Life Technologies), according to the manufacturer’s instructions. DiIC18-labeled T-APCs were used as targets for specified T cell clones. To enable separate gating of CD8^+^T-APC and effector CTL, the latter were surface-biotinylated with Biotin-7-NHS (Roche) according to the manufacturer’s instructions. CD8^+^-T-APCs were co-cultured with effector CTL either immediately or 6-, 24- or 48-hours post separation with anti-human CD8 particles, as specified in each experiment.

### Trogocytosis assay and functional analysis of effector CTL

Trogocytosis assays were performed as previously described, with minor modifications. Briefly, DiIC18-labeled CD8^+^T-APC were co-cultured with surface-biotinylated effector CTL (1–5 × 10^5^ lymphocytes/well in 200 μl final volume) in U-shaped 96-well plates at a 1:1 effector-to-target ratio. Following incubation for 1 hour at 37°C, cells were washed twice with PBS containing 0.5 mM EDTA to ensure conjugate dissociation, and re-suspended in FACS buffer consisting of PBS supplemented with 0.5% BSA and 0.1% NaN3. The cells were stained with FITC-conjugated anti-CD8 antibodies and APC-conjugated streptavidin (both from eBioscience) for 30 min at 4°C and acquired using a BD LSRII flow cytometer. The capacity of effector CTLs to perform trogocytosis was defined as percent of streptavidin^+^ DiIC18^+^ double-positive cells, gated on CD8^+^ lymphocytes, following exclusion of doublets. Cytolytic activity of effector CTLs was measured by surface CD107A staining with anti—CD107A-FITC antibodies (eBioscience).

For intracellular cytokine staining, the Cytofix/Cytoperm Plus Kit with GolgiPlug (BD) was used, according to the manufacturer’s recommendations. Briefly, 1–5 × 10^5^ effector CTLs were co-cultured with CD8^+^T-APC for 6 hours at a 1:1 ratio, as described above. Brefeldin A (1 μg/ml, GolgiPlug) was added for the last 4 h to enable intracellular protein accumulation. The cells were harvested, re-suspended in FACS buffer and surface stained with APC-conjugated streptavidin (eBioscience) for 30 min at 4°C, to enable gating on the biotinylated effector CTL population. Following fixation and permeabilization, intracellular cytokines were stained with one of the following mAb: anti—IFN-γ-PE or FITC, anti—TNF-α-PE (all from eBioscience) or anti—TNF-α- Pacific blue (Biolegend). Appropriate isotype controls were used. Acquisition was done using a BD LSRII flow cytometer. The data were analyzed using FCS Express software (De Novo, Los Angeles, CA).

### Detection of acquired pMHC on murine T-APC and proliferation assay

OT-I spleen cells (1–3x10^5^) were co-cultured with EG7 cells at a 1:1 ratio for 1 hour at 37°C. EL4 cells were used as a control target. The cells were harvested, washed twice with FACS buffer and stained with biotinylated-anti Ova_257–264_/H-2Kb (clone 25-D1.16, eBioscience). Surface pMHC on CD8^+^ T cells was detected by flow cytometry following concomitant staining with anti-CD8-FITC and streptavidin-APC (both from eBioscience).

To measure effector CTL proliferation, OT-I spleen cells were co-cultured with EG7 or EL4 cells, as described above. CD8^+^ T cells (T-APC) were purified using BD IMag anti-mouse CD8 particles (BD Biosciences) and labeled with eFluor 450 proliferation dye according to the manufacturer’s instructions (eBioscience). eFluor 450-labeled T-APC were co-cultured at a 1:1 ratio for 3 days with naïve OT-I CD8^+^ T cells (effector CTL) labeled with carboxyfluorescein succinimidyl ester (CFSE, Life Technologies). The cells were harvested, washed and stained with anti-mouse CD8-allophycocyanin (eBioscience) followed by flow cytometry analysis of CFSE dilution in effector CTL (CD8^+^eFluor450^-^). Proliferation of OT-I CD8^+^ T cells, treated with 2.5 μg/ml Concanavalin A (ConA) was used as a positive control.

### Caspase-3 cleavage CTL assay

Intracellular staining of cleaved caspase-3 was performed as described [28]. Briefly, T-APC (produced by co-culture of MART-1_26–35_-specific T cells with 624*mel* melanoma cells) or non T-APC (produced by co-culture of MART-1_26–35_-specific T cells with M171 melanoma cells) were stained with DDAO-SE cell tracker (Life Technologies) according to the manufacturer’s protocol. Following repeated washing with PBS, DDAO-SE-labeled target cells were co-cultured with either MART-1_26–35_-specific or MUC1-specific effector CTL for 1 hour at a 1:1 ratio. The cells were then harvested and washed twice with PBS containing 1% BSA. Following fixation and permeabilization according to the eBioscience protocol, intracellular cleaved caspase-3 was labeled with rabbit anti-cleaved caspase-3-PE (BD Pharmingen) for 30 min at 4°C. Appropriate isotype control was used. Cells were washed with permeabilization buffer, re-suspended in FACS buffer, subjected to flow cytometry and analyzed as above.

### Confocal microscopy and live cell imaging

To visualize cell-cell interactions between the lymphocytes, 2E2 cells used as CD8^+^T-APC or as effector CTL were pre-labeled with the lipophilic dyes PKH67 and PKH26 (Sigma) respectively, according to the manufacturer’s instructions. Labeled CD8^+^T-APC and effector CTL were co-incubated in a chambered cover glass (Thermo Scientific, Germany) for 1 hour at 37°C. Images were obtained with a Zeiss LSM 510 META confocal microscope (Zeiss, Jena, Germany) and analyzed using LSM510 software version 3.2 (Zeiss).

## Results

### CD8^+^T-APC can activate anti-tumor CD8 T cells of the same antigen specificity

**Fig 1:**
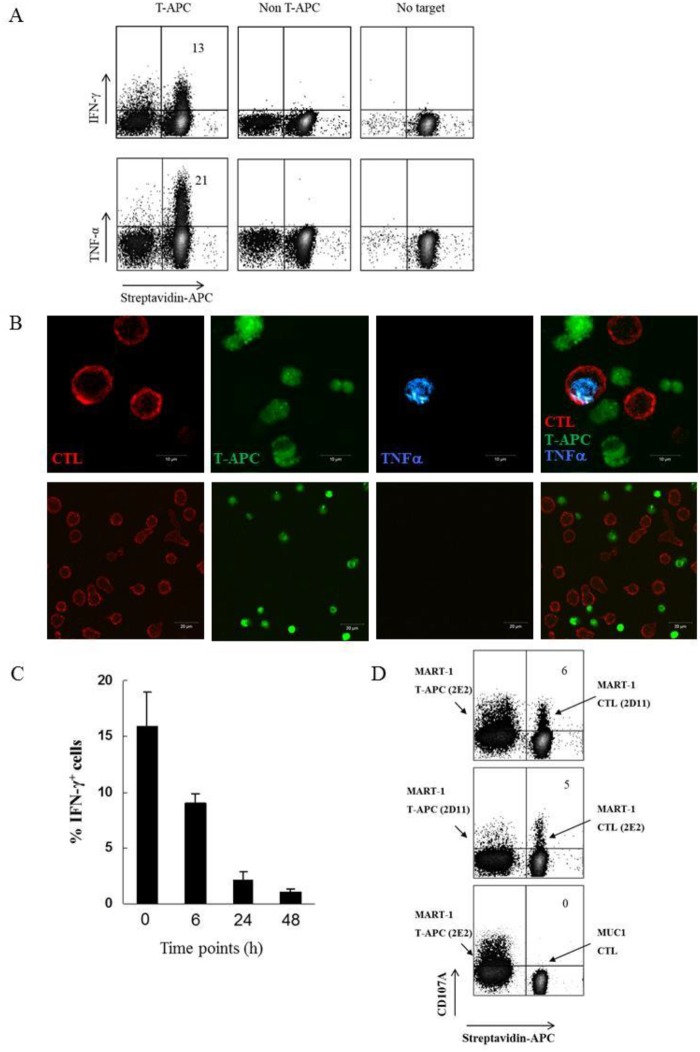
CD8^+^T-APCs activate anti-tumor CD8 T cells of the same antigen specificity. **(A-C)** Cytokine production by effector CTLs in response to activation by T-APCs. **(A)** DiIC_18_-labeled CD8^+^T-APCs (see materials and methods) were incubated for 6 hours with surface-biotinylated effector CTLs. Cytokine-producing effector CTLs were defined based on intracellular IFN-γ or TNF-α staining of CD8^+^streptavidin^+^ lymphocytes. Numbers in upper right quadrants indicate the percentage of IFN-γ ^+^ (upper panel) or TNF-α ^+^ (lower panel) effector CTLs. Labels indicate cells used as targets for CTLs: CTLs co-cultured with 624*mel* melanoma cells are designated T-APC; CTLs co-cultured with irrelevant M171 melanoma cells are designated non T-APC. **(B)** Confocal images of cytokine-producing effector CTLs. Calcein AM labeled CD8^+^T-APCs (*green*, upper panel) or non T-APCs (*green*, lower panel) were co-cultured for 6 hours with streptavidin-allophycocyanin-stained effector CTLs (*red*). Intracellular TNF-α production (*blue*) by effector CTLs is shown. Scale bars are 10 μm (upper panel) and 20 μm (lower panel). **(C)** Time period that CD8^+^T-APCs activate effector CTLs. Effector CTLs were co-cultured with CD8^+^T-APCs either immediately or 6, 24 and 48 hours after CD8^+^T-APC purification. Data are mean ± SE (n = 3 replicates/group) percentage of IFN-γ ^+^ effector CTLs, gated on CD8^+^ T cells. **(D)** CD8^+^T-APCs trigger degranulation of effector CTLs. CD8^+^T-APCs were generated as described above (1A) and co-cultured with effector CTLs. Cytolytic activity of T cells was measured by detection of surface CD107A on CD8^+^streptavidin^+^ effector CTLs. Number in upper right quadrants indicates the percentage of CD107A^+^ streptavidin^+^ effector CTLs. Data are representative of at least three independent experiments.

Trogocytosis between melanoma cells and cytotoxic T lymphocytes (CTLs) imprint the latter with membrane fragments containing tumor antigens [23,24]. We previously showed that the ability of CTL clones to perform trogocytosis reflects the strength of their anti-tumor reactivity [25]. Now we asked whether these CTLs, following exposure to melanoma cells, have the ability to present antigen to neighboring CD8^+^ T cells, thus acting as “CD8^+^T-APC”. To this end, the MART-1_27–35_-reactive clone 2E2 was co-cultured with cognate (624*mel)* melanoma, purified, labeled with membrane dye and then co-incubated with 2E2 cells (labeled with a different membrane dye) that served as CTLs. In this experimental setting, CD8^+^T-APCs and effector CTLs were easily distinguishable based on the two different colors. CTLs were examined for cytokine production and degranulation. The results, presented in Fig. 1A, show that after co-incubation with CD8^+^T-APCs, 13% and 21% of CTLs produced IFN-γ and TNF-α, respectively. No cytokine-producing CTLs were detected following co-culture with control CD8^+^ *non* T- APCs generated by co-incubation of 2E2 cells with non-cognate melanoma (M171). In order to confirm that cytokines were produced by effector CTLs, CD8^+^T-APC-induced cytokine production was also visualized by confocal microscopy. After 6 hours of co-culture with CD8^+^T-APCs (Fig. 1B, upper panel) (but not with non T-APCs, lower panel), TNF-α clearly accumulated within effector CTLs.

In order to determine how long CD8^+^T-APCs are able to present acquired tumor components, fresh and 6 hour-old CD8^+^T-APCs were co-cultured with effectors. As a result, IFN-γ-production was triggered in 16% and 9% of the CTLs, respectively (Fig. 1C). Although 24 hour-old and even 48 hour-old CD8^+^T-APCs showed residual ability to stimulate CTLs (2.1% and 1.1%, respectively), these could also represent background levels.

The effect of CD8^+^T-APCs on the cytolytic activity of CTLs was measured by surface expression of CD107A (LAMP-1), a surrogate marker of lysosomal degranulation [24,29]. As shown in Fig. 1D, 5 and 6% of CTLs of two different clones exhibited cytolytic activity in response to CD8^+^T-APCs, further demonstrating the ability of trogocytosis-generated APCs to activate tumor-specific CD8^+^ T cells. No cytolytic activity was detected when non-melanoma-reactive, HLA-A2^+^, MUC-1 CTLs were co-incubated with melanoma-entrained CD8^+^T-APCs.

To exclude the possibility that non-specific adherence of melanoma fragments, rather than CD8^+^T-APCs, account for CTL activation, we co-cultured MUC-1-reactive CD8^+^ T cells with 624*mel*. After separation of the lymphocytes from the melanoma (which they do not recognize), MART-1-reactive effector CTLs were added. No CTL activation was observed, thus excluding melanoma contamination (S1 Fig.). Collectively, these data demonstrate that tumor-specific CD8^+^ T cells can activate other CTLs of the same antigen specificity, by re-presentation of tumor derived antigens acquired through trogocytosis.

Next, CD8^+^ T cells were non-specifically stimulated in a TCR-dependent (by anti-CD3 mAb) or independent (by PMA/ionomycin) manner, and examined for their ability to act as CD8^+^T-APCs. Under both conditions, the activated lymphocytes failed to stimulate effector T cells (S2 Fig.).

### CD8^+^T-APCs can activate tumor-reactive CTL clones of different antigen specificity

**Fig 2:**
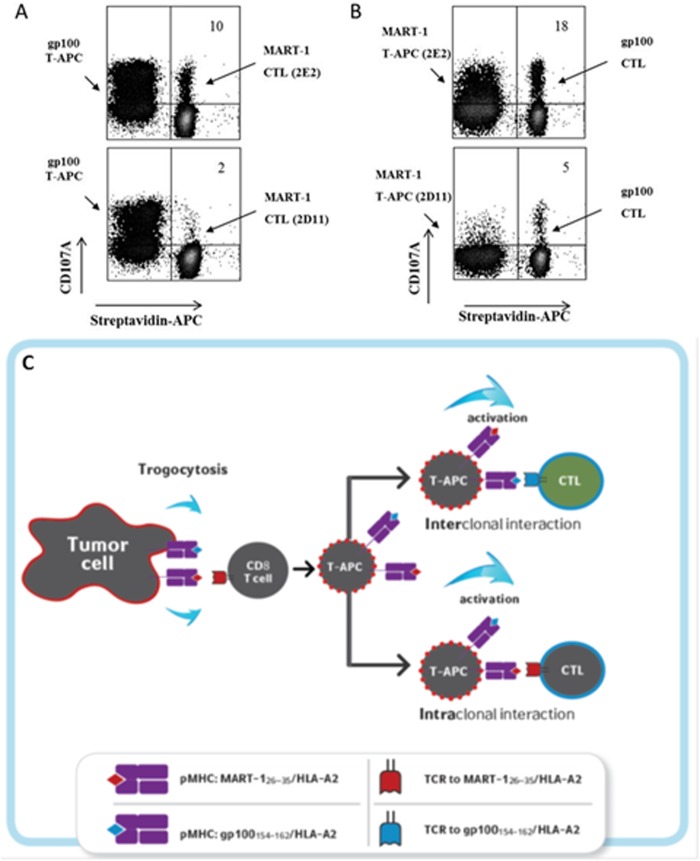
CD8^+^ T-APCs induce degranulation of effector CTLs with different antigen specificity. (**A, B**) CD8^+^ clones with different antigen specificity were used as CD8^+^T-APCs and effector CTLs. Biotinylated effector CTLs were co-cultured with CD8^+^T-APCs, stained with anti-CD107A mAb and streptavidin-allophycocyanin and analyzed by flow cytometry. (**A**) The gp100_154–162_-specific clone 1G2 was used as CD8^+^T-APC for the MART-1_26–35_-specific CTL clones (2E2 and 2D11). **(B)** The MART-1_26–35_-specific clones 2E2 and 2D11 were used as CD8^+^T-APC for the gp100_154–162_-specific clone (1G2). Numbers in upper right quadrants indicate the percentage of CD107A^+^streptavidin^+^ lymphocytes, gated on the CD8^+^ population (effector CTLs). Data are representative of three independent experiments. (**C**) Graphic presentation of intra- and inter-clonal T cell cross talk.

Above, we described the ability of CD8^+^T-APCs to present acquired tumor antigens to CTLs expressing the *same* TCR. Following, we asked whether CD8^+^T-APCs could activate CTLs with *different* TCRs, thus leading to an *inter*-clonal T cell response. To investigate this possibility, MART-1 clones (2E2 or 2D11) were used as effector CTLs, and a gp100-specific clone (1G2) as CD8^+^T-APC. As shown in Fig. 2A, gp-100-specific CD8^+^T-APCs triggered degranulation of the MART1-specific CTLs. In line with the trogocytosis capacity of the clones [25], 2E2 (a CD8^+^ T cell clone with high functional capacity) was more efficient than 2D11 (low functional capacity) (10% versus 2%). The ability of CD8^+^T-APCs to activate different antigen-specific CTL clones was further demonstrated by changing the roles of the clones, with MART-1-reactive clones now the CD8^+^T-APC, and the gp100-reactive clone the effector CTL (Fig. 2B). Apparently, the stimulation of cells with different antigen specificity (2B) is stronger than stimulation of cells with the same specificity (1D). However, a conclusion cannot be drawn from the evaluation of two antigens. Fig. 2C is a graphic presentation of intra- and inter-clonal activity. Taken together, these results provide evidence that, following encounter with tumor, melanoma-reactive T cells present a variety of antigens and activate CTLs of different specificity.

### Following trogocytosis, CD8^+^T-APCs display peptide-MHC complexes

In the previous sections we used nonspecific membrane dyes to detect CD8^+^T-APCs. The ability of these antigen presenting cells to activate CTLs suggested that intact pMHCs were part of the membrane transferred. In order to prove this, we used murine OT-I T cells, whose MHC-conjugated peptide Ova_257–264_ can be detected by a specific antibody.

**Fig 3:**
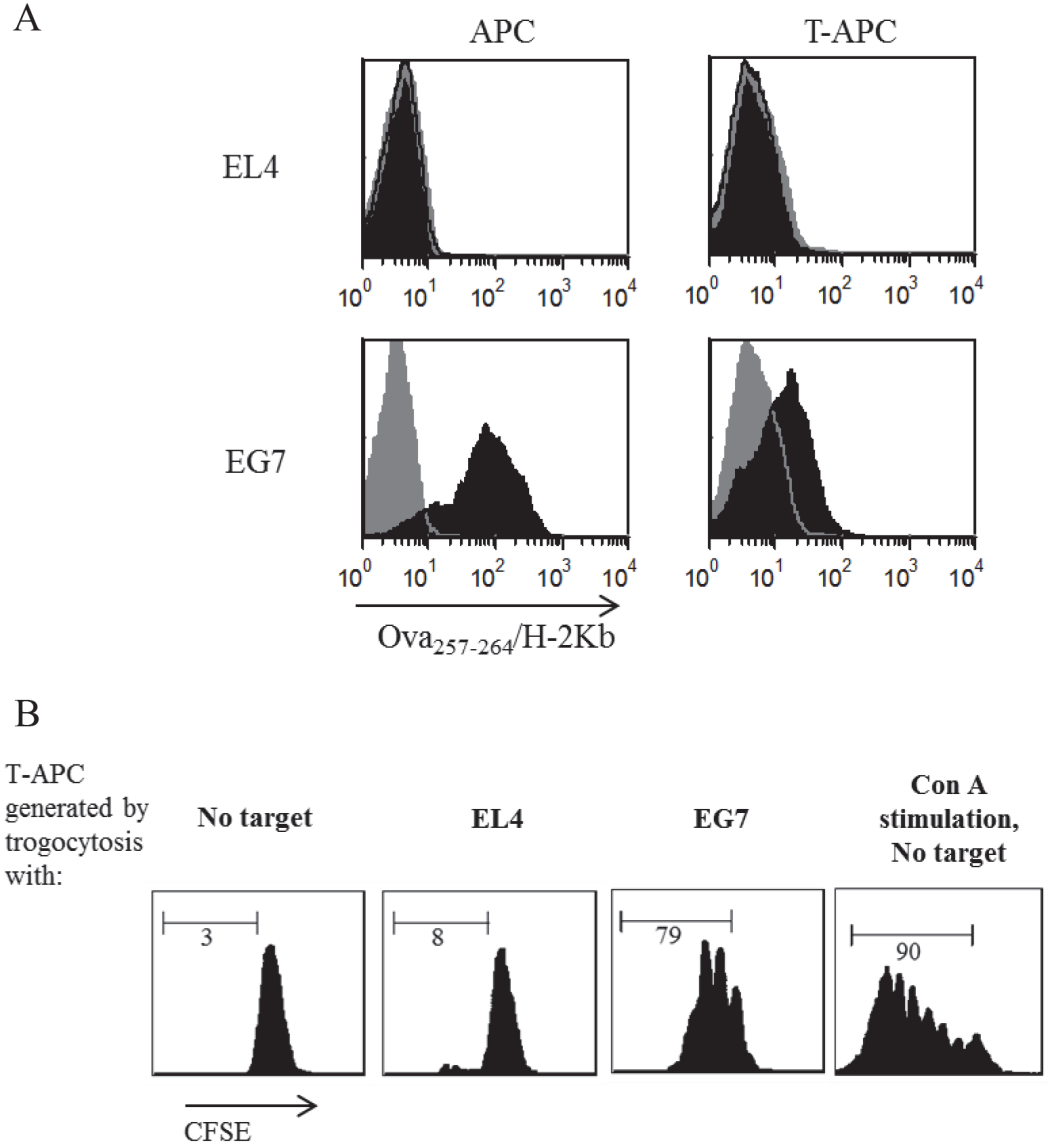
The effect of CD8^+^T-APCs on effector CTLs is mediated by tumor-derived pMHC. **(A)** Ova-expressing EG7 and parental EL4 target cell lines (*Left column*) and target-entrained CD8^+^T-APC (generated following co-culture of OT-I CD8^+^ T cells with designated targets, *right column*) were labeled with Ova_257–264_/H-2Kb-specific mAb (*black histogram*). *Grey histogram*, background staining with isotype control antibody. **(B)** Proliferation of OT-I CD8^+^ T cells stimulated with CD8^+^T-APCs. CFSE-labeled OT-I T cells were left untreated (no target) or co-cultured with the following T-APCs: OT-I CD8^+^ pre-incubated with EL4 (EL4) or OT-I CD8^+^ pre-incubated with EG7 cells (EG7). ConA stimulation was used as positive control (right). *Scale bars*, proliferating lymphocytes that divided at least twice. *Numbers*, percentage of dividing CD8^+^ T cells. Data are representative of two independent experiments.

To generate CD8^+^T-APCs, OT-I cells were co-cultured with EG7 cells (ovalbumin-expressing EL4 thymoma). Kb-Ova_257–264_ pMHC was detected on EG7 by staining with pMHC-specific antibody [30] (Fig. 3A, left column), and was clearly detected on CD8^+^T-APCs following co-culture with EG7 targets (Fig. 3A, right column). In contrast, no Kb-Ova_257–264_ staining was detected on OT-I cells when co-cultured with parental EL4.

Following, we evaluated the functionality of pMHC captured and presented by CD8^+^T-APCs. To this end, we monitored proliferation of CFSE-labeled OT-I CTLs co-cultured with CD8^+^T-APCs. As shown in Fig. 3B, CD8^+^T-APCs triggered proliferation of OT-I CTLs, detected in CTLs cultured with CD8^+^T-APC (generated by co-incubation with EG7 cells) or with ConA (positive control), but not with non-T-APCs (generated by co-incubation with EL4 cells). Thus, we show by direct staining that intact pMHCs are present on the CD8^+^T-APCs after trogocytosis, endowing them with the capacity to induce CTL proliferation.

### CD8^+^T-APCs interact with effector CTLs resulting in secondary trogocytosis

**Fig 4:**
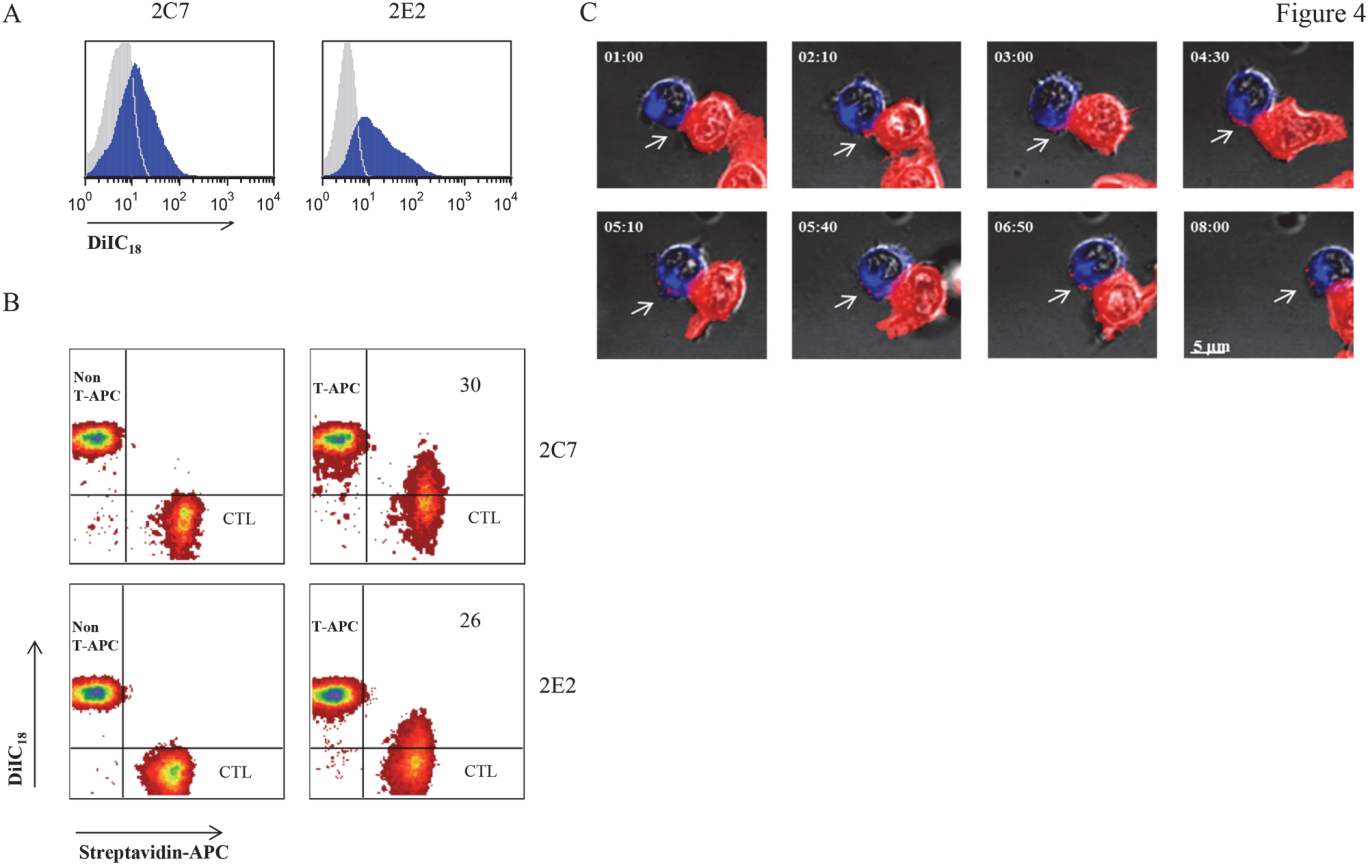
CD8^+^T-APC induce secondary trogocytosis by tumor specific CTL. **(A, B)** CD8^+^T-APCs and non-T-APCs were generated by co-incubation with cognate or irrelevant melanoma, respectively. They were then sorted, labeled with DiIC18 (see Methods) and co-cultured with surface biotinylated 2C7 or 2E2 clones (effector CTL). The culture was then stained with anti-CD8 antibodies and streptavidin- allophycocyanin, and subjected to flow cytometry. **(A)** Histograms indicate secondary trogocytosis by the presence of DiIC18 on 2C7 or 2E2 CTLs, gated on CD8^+^streptavidin^+^ populations, following co-culture with CD8^+^T-APC (*blue*) or non-T-APC (*grey*). **(B)** Secondary trogocytosis was measured by presence of DiIC18 on the CD8^+^streptavidin^+^ population (effector CTL) and streptavidin-allophycocyanin on the CD8^+^DiIC18^+^ population (*left column*, non-T-APC, *right column*, T-APC). Numbers in upper right quadrants indicate the percentage of DiIC18- stained effector CTL (performing secondary trogocytosis). **(C)** PKH67-labeled CD8^+^T-APCs (*red*) were co-cultured with PKH26-labeled effector CTLs (*blue*). The lymphocytes were co-incubated in a chambered cover-glass and subjected to confocal microscopy. A snapshot series of 8 min is presented. *Arrows*, transfer of membrane fragments (secondary trogocytosis) from CD8^+^T-APC to CTL. Scale bars, 15 μm. Data are representative of at least five independent experiments (A, B) or of three experiments (C).

To evaluate the possibility of serial membrane transfer from lymphocyte to lymphocyte, we set to measure and visualize secondary trogocytosis. For this purpose we used effector CTLs and CD8^+^T-APCs stained with different membrane dyes. As shown in Fig. 4A, two melanoma-reactive T cell clones (2C7 and 2E2) captured membrane fragments from CD8^+^T-APCs, as shown by the presence of the DiIC18, used for CD8^+^T-APCs labelling, on the T cell clones (*blue* histogram). No DiIC18 presence was measured when the T cell clones were cultured with non T-APCs (*grey* histogram). Fig. 4B demonstrates that the transfer of membrane from CD8^+^T-APCs to effector CTLs (secondary trogocytosis) is unidirectional, as only tumor-entrained CTLs (CD8^+^T-APCs) can serve as membrane donors (Fig. 4B). Next, we used live cell microscopy to follow the sequence of events taking place during secondary trogocytosis. The entire uninterrupted image sequence is available as a S1 Movie. The snapshot photos presented in Fig. 4C provide a close-up view of the focal interaction between a single CD8^+^T-APC and an effector CTL during 8 minutes of live cell imaging. At 1 minute, a blue-labeled CTL and a red-labeled CD8^+^T-APC established a tight contact zone with a marked increase in stain intensity, suggesting formation of an immunological synapse between the cells. The next three images document the transfer of membrane fragments from the CD8^+^T-APC to the CTL. At 5 minutes, the transferred fragments appear detached from the CD8^+^T-APC. Three single spots are visualized on the recipient cell at sites distal to the immune synapse. Consistent with flow cytometry results, there was no evidence for secondary trogocytosis by effector CTL following co-culture with non-CD8^+^T-APC (4B and data not shown). Taken together, these results show that antigen-specific CD8^+^ T cells imprinted with tumor membrane fragments interact with effector CTLs, resulting in secondary trogocytosis. The fast kinetics of immune synapse formation and secondary trogocytosis, 1 and 5 minutes respectively, reflect the efficiency of lymphocyte-to-lymphocyte membrane transfer.

### Acquired tumor antigens mark CD8^+^T-APC as targets for fratricide by tumor-specific CTLs

**Fig 5:**
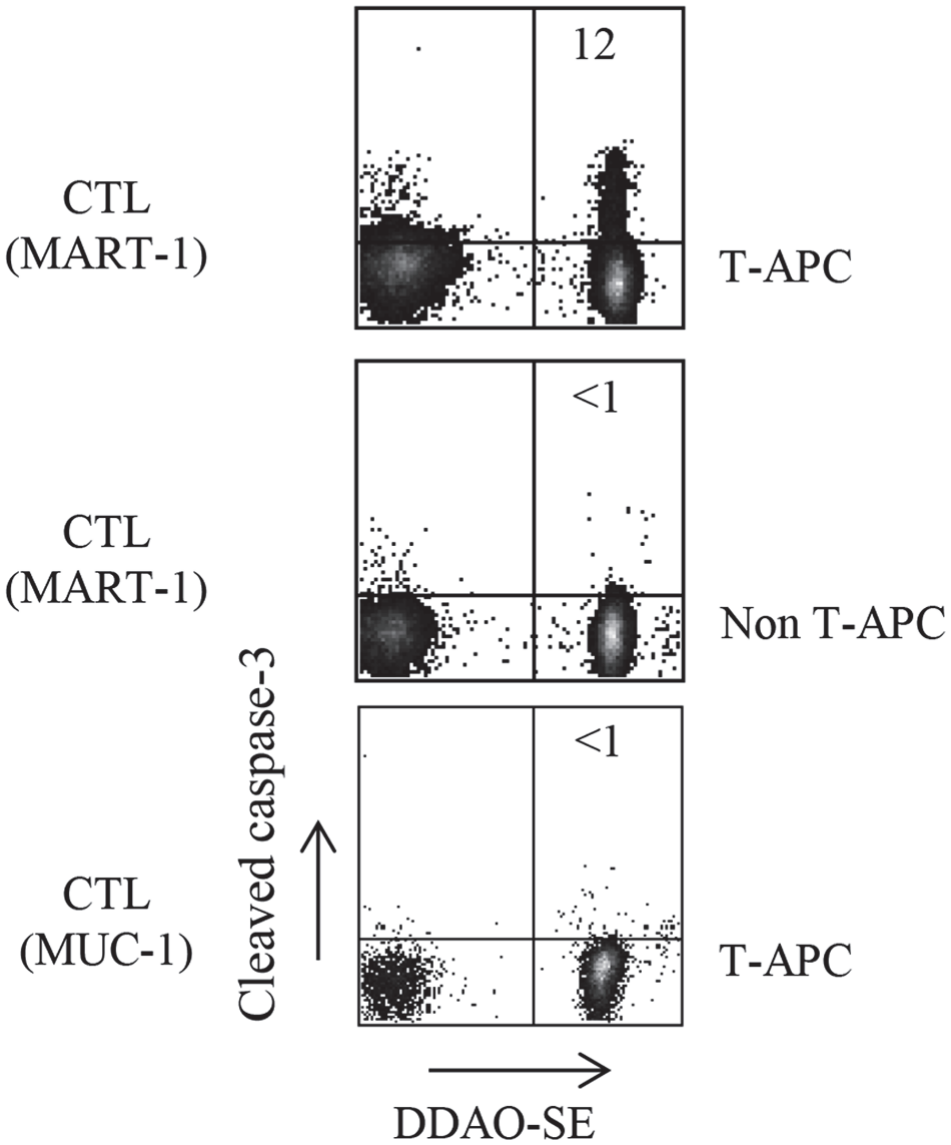
Assessment of CTL fratricide activity based on detection of cleaved caspase-3. MART-1- or MUC-1- reactive CTLs were co-cultured with DDAO-SE-tagged CD8^+^T-APCs and non T-APCs, generated by co-incubation of 2E2 cells with 624*mel* and M171 melanoma cells, respectively. T-APC damage was examined based on detection of intracellular cleaved caspase-3 in the DDAO-SE^+^CD8^+^ population. Numbers in upper right quadrants indicate the percentage of cleaved caspase 3-positive CD8^+^T-APC cells. Data are representative of three independent experiments.

Trogocytosis of tumor-derived antigens enables CD8^+^ T cells to activate other CTLs (Figs. 1 and 2), but may also mark CD8^+^T-APCs for killing by tumor-reactive CTLs (fratricide) [31]. To verify this possibility, CD8^+^T-APCs were used as targets for CTLs. Killing of CD8^+^T-APCs was examined by flow cytometry for cleaved caspase-3, an early marker of apoptosis, in the damaged cells [28]. We compared CTL activity against CD8^+^T-APCs versus non T-APCs. As shown in Fig. 5, only CD8^+^T-APCs displayed caspase-3 cleavage as an indicator of CTL-induced damage (upper plot), whereas non T-APCs showed no cleavage (middle plot). CD8^+^T-APC susceptibility to CTL-induced apoptosis was antigen-dependent, since MUC-1-specific CTLs did not kill the CD8^+^T-APC (lower plot). Hence, CD8^+^ T cells that are capable of activating bystander CTLs via presentation of acquired tumor antigens are concomitantly susceptible to fratricide by tumor-reactive CTLs.

## Discussion

In the present study we provide evidence for functional tumor antigen presentation by CD8^+^ T cells. This activity of effector T cells adds a new dimension to the concept of functional plasticity of the immune response in the context of anti-cancer immunity [31]. Following encounter with tumor cells, CD8^+^ T cells capture tumor antigens by trogocytosis, a mode of membrane fragment transfer between immune cells [2]. Here we show that, after interaction with the tumor, cognate CD8^+^ T cells are endowed with antigen presenting ability, capable of activating neighboring CTLs. We designated these cells CD8^+^T-APCs. Three major findings support the identification of tumor-entrained CD8^+^ T cells as APCs: 1. They establish a physical immune synapse with effector CTLs (Fig. 4); 2. They stimulate effector CTLs, as measured by cytokine secretion and degranulation (Fig. 1); 3. They trigger naïve CTLs to proliferate in an antigen-specific manner (Fig. 3).

Although the functional impact of antigen acquisition and re-presentation among immune cells has been the subject of intense investigation (reviewed in [12][32]), the biological consequences of *tumor* antigen re-presentation in T-cell: T-cell cross talk received less attention. In the context of cancer, it was tumor-derived molecules with immune modulatory properties that were identified in the recipient lymphocytes. Examples include PD-L1, HLA-G and CD86, shown to induce immunosuppression in NK and CD8^+^ cells following their cross-dressing with membranes taken from melanoma or multiple myeloma [24,33,34]. Although not an immunomodulator *per se*, oncogenic H-RAS was shown to relocate from melanoma to lymphocytes and induce autonomous signaling, eventually leading to enhanced activity [35].

Only recently has the phenomenon of uptake of pMHC molecules from tumor cell membrane by CTLs via trogocytosis been acknowledged [36]. The emphasis, however, was on the effect of depletion of pMHCs from the tumor surface, snatched by low avidity CTLs, leading to insufficient triggering of high-avidity CTLs and diminished melanoma lysis. The present report is in accord with our previous finding regarding the imprinting of melanoma-specific CTLs with multiple—cognate and non-cognate- melanoma antigens [24]. Here we took this observation one step further, to shed light on the role played by trogocytosis in the capacity of CD8^+^ T cells to present tumor antigens. The picture emerging from our data is that encounter with the tumor enables reactive T cells to acquire a variety of tumor antigens and spread the activation cascade within their own clone or to other T cells, thus amplifying the process of epitope spreading [35].

These results demonstrate an alternative mode of tumor antigen presentation, adding to the already recognized pathways of direct (by tumor cells) and indirect (by professional APC) presentation. This conclusion was further corroborated using OT-I CD8^+^ T cells to enable direct pMHC detection and thus providing direct proof that specific T cell activation is conferred by the transferred epitope linked to an MHC (Fig. 3).

There is a yin-yang aspect to antigen presentation by tumor experienced CD8^+^T-cells. On the one hand, CD8^+^T-cells can activate neighboring lymphocytes; on the other hand, the presence of tumor-derived molecules on their surface marked CD8^+^T-cells as targets for fratricide by other tumor-reactive CTLs (Fig. 5). The involvement of trogocytosis in the elimination of activated NK cells and lymphocytes has been described and proposed as a regulatory mechanism to prevent hyper-responsiveness [37,38]. Our findings extend this concept to anti-tumor immune responses, which often include CTLs directed against self-antigens [39], and may thus represent a mechanism to attenuate autoimmune attack.

In summary, we show that tumor-specific CD8 T lymphocytes can propagate the immune response by activation of additional T cells through intra- and inter-clonal interaction, and at the same time can render themselves susceptible to fratricide. We suggest that the ability of anti-cancer effector lymphocytes to modulate the CTL response may represent a novel mechanism of immune plasticity.

## Supporting Information

S1 Table Characterization of lymphoid cell lines and clones. (TIF)

S1 Fig Non melanoma-reactive CTLs do not activate a MART-1-reactive clone. MUC-1_63–71-_specific CD8^+^ T cells were co-cultured with 624*mel*, sorted using positive selection with magnetic particles and evaluated as CD8^+^T-APC for MART-1-specific 2E2 lymphocytes, pre-labeled with surface biotin. Following co-culture for 1 hour at 37°C, cytolytic activity of effector CTLs was measured by detection of surface CD107A on streptavidin-APC-positive 2E2 cells.
(TIF)

S2 Fig Degranulation of CTLs upon non-specific activation or co-culture with activated CTL. **(A)** 2E2 T cells were activated by PMA/ionomycin or plate-bound anti-CD3/CD28 antibodies. Following activation, CTLs were double-stained with anti-CD8 and anti-CD107A antibodies and analyzed by flow cytometry. *Grey histogram*, background staining with isotype control mAb; *Black histogram*, staining with anti-CD107A mAb gated on CD8^+^ cells. (B) 2E2 T cells were activated by PMA/ionomycin or anti-CD3/CD28 antibodies and examined as CD8^+^T-APC for biotin-labeled resting 2E2 cells, used as effector CTL. Following 1 hour co-culture, the cells were stained with anti-CD107A mAb and streptavidin and analyzed by flow cytometry. The cytolytic activity of effector CTL was measured by detection of CD107A on the streptavidin^+^ population. *Grey histogram*, CD107A staining of effector CTL only; *Black histogram*, CD107A staining of effector CTL co-cultured with activated CD8^+^ T cells.
(TIF)

S1 Movie Visualization of cell-cell interactions between lymphocytes. 2E2 cells used as CD8^+^T-APC or as effector CTL were pre-labeled with the lipophilic dyes PKH67 and PKH26 respectively, and co-incubated in a chambered cover glass for 1 hour at 37°C.
(AVI)
