# Supplementary figures and images for: Human T Cell Crosstalk Is Induced by Tumor Membrane Transfer
Source: PLoS One. 2015 Feb 11;10(2):e0118244. doi: 10.1371/journal.pone.0118244 (PMC4324967; doi:10.1371/journal.pone.0118244)

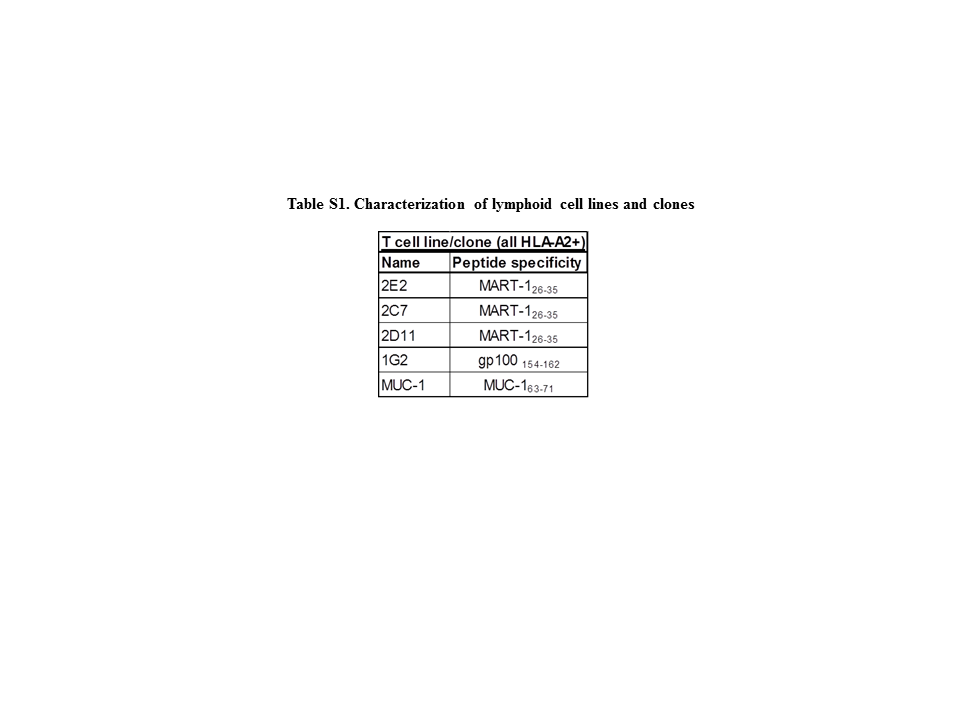

Supplement: S1 Table — (TIF) [file pone.0118244.s001.tif]

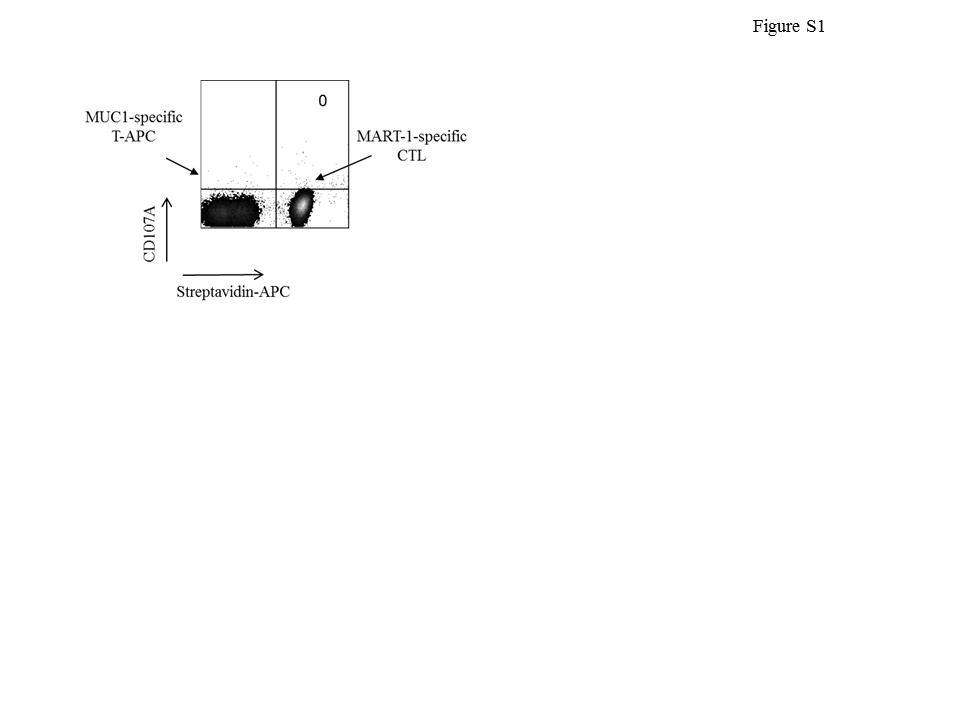

Supplement: S1 Fig — MUC-163–71-specific CD8+ T cells were co-cultured with 624mel, sorted using positive selection with magnetic particles and evaluated as CD8+T-APC for MART-1-specific 2E2 lymphocytes, pre-labeled with surface biotin. Following co-culture for 1 hour at 37°C, cytolytic activity of effector CTLs was measured by detection of surface CD107A on streptavidin-APC-positive 2E2 cells. (TIF) [file pone.0118244.s002.tif]

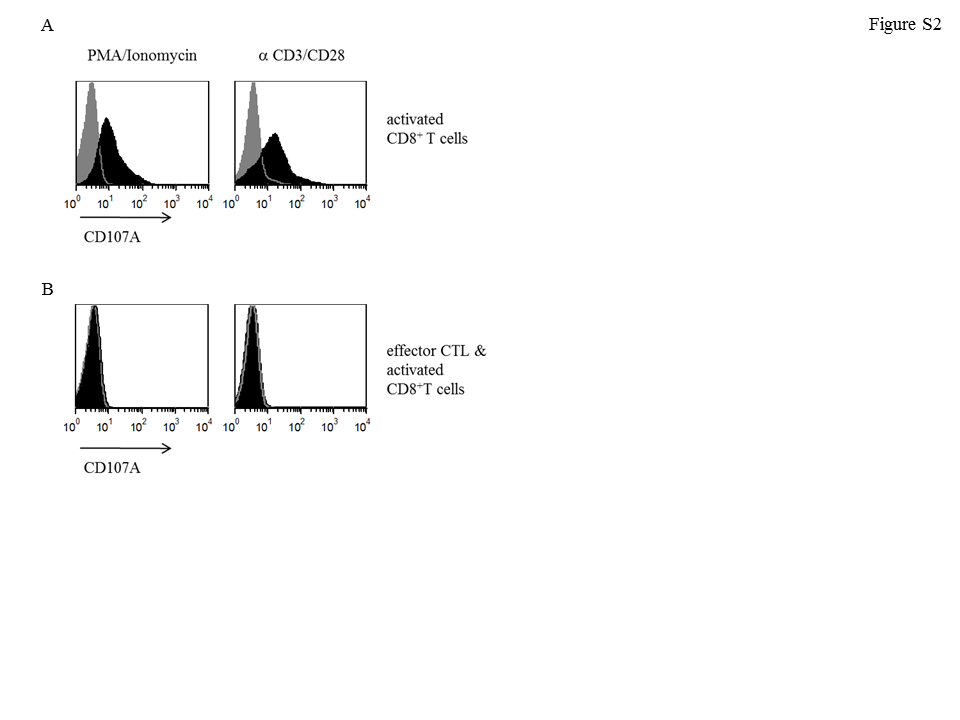

Supplement: S2 Fig — (A) 2E2 T cells were activated by PMA/ionomycin or plate-bound anti-CD3/CD28 antibodies. Following activation, CTLs were double-stained with anti-CD8 and anti-CD107A antibodies and analyzed by flow cytometry. Grey histogram, background staining with isotype control mAb; Black histogram, staining with anti-CD107A mAb gated on CD8+ cells. (B) 2E2 T cells were activated by PMA/ionomycin or anti-CD3/CD28 antibodies and examined as CD8+T-APC for biotin-labeled resting 2E2 cells, used as effector CTL. Following 1 hour co-culture, the cells were stained with anti-CD107A mAb and streptavidin and analyzed by flow cytometry. The cytolytic activity of effector CTL was measured by detection of CD107A on the streptavidin+ population. Grey histogram, CD107A staining of effector CTL only; Black histogram, CD107A staining of effector CTL co-cultured with activated CD8+ T cells. (TIF) [file pone.0118244.s003.tif]
